# MiRNAs and Muscle Regeneration: Therapeutic Targets in Duchenne Muscular Dystrophy

**DOI:** 10.3390/ijms22084236

**Published:** 2021-04-19

**Authors:** Amelia Eva Aránega, Estefanía Lozano-Velasco, Lara Rodriguez-Outeiriño, Felicitas Ramírez de Acuña, Diego Franco, Francisco Hernández-Torres

**Affiliations:** 1 Department of Experimental Biology, Faculty of Experimental Sciences, University of Jaen, Paraje Las Lagunillas s/n, 23009 Jaen, Spain; evelasco@ujaen.es (E.L.-V.); lrodrigu@ujaen.es (L.R.-O.); fracuna@ujaen.es (F.R.d.A.); dfranco@ujaen.es (D.F.); fraheto@ujaen.es (F.H.-T.); 2 Medina Foundation, Technology Park of Health Sciences, Av. del Conocimiento 34, 18016 Granada, Spain; 3 Department of Biochemistry and Molecular Biology III and Immunology, Faculty of Medicine, University of Granada, Avda. de la Investigación 11, 18016 Granada, Spain

**Keywords:** microRNA, satellite cell, myogenesis, muscle regeneration, muscular dystrophies

## Abstract

*microRNAs* (*miRNAs*) are small non-coding *RNAs* required for the post-transcriptional control of gene expression. MicroRNAs play a critical role in modulating muscle regeneration and stem cell behavior. Muscle regeneration is affected in muscular dystrophies, and a critical point for the development of effective strategies for treating muscle disorders is optimizing approaches to target muscle stem cells in order to increase the ability to regenerate lost tissue. Within this framework, miRNAs are emerging as implicated in muscle stem cell response in neuromuscular disorders and new methodologies to regulate the expression of key microRNAs are coming up. In this review, we summarize recent advances highlighting the potential of miRNAs to be used in conjunction with gene replacement therapies, in order to improve muscle regeneration in the context of Duchenne Muscular Dystrophy (DMD).

## 1. Introduction

One-half of the body’s mass is composed of skeletal muscles that have important functions basic to life, such as locomotion, postural behavior and breathing. Hence, the maintenance of a working skeletal musculature is crucial for the proper functioning of the body. Fortunately, the adult mammalian skeletal muscle is one of the few tissues capable of efficient regeneration in response to injury or damage. This ability is due, at least partly, to a population of undifferentiated mononuclear myogenic progenitor cells, known as satellite stem cells [1], which reside between the basal lamina and sarcolemma of myo-fibers. Although satellite cells (SCs) are mitotically quiescent, they activate in response to injury or increased contractile activity, thereby re-entering into the cell cycle, proliferating, differentiating, fusing and, finally, regenerating myofibers [2,3]. Genetic dissection of this process has revealed that developmental pathways required for embryonic myogenesis also regulate muscle regeneration [4,5]. Importantly, activated SCs also undergo self-renewal to restore the pool of quiescent SCs that are able to support additional rounds of regeneration [5,6]. SCs activation and differentiation are also controlled by mitogenic factors released by inflammatory cells during the early stages of the muscle regeneration process [7,8].

Muscle regeneration is affected in muscle-wasting diseases. It is worth highlighting that, in muscular dystrophies such Duchenne Muscular Dystrophy (DMD), progressive muscle wasting and weakness is often associated with an exhaustion of muscle regeneration potential. Therefore, the progressive loss of muscle mass has been attributed, at least partly, to the inability of muscle stem cells to efficiently regenerate tissue lost as the result of the disease [9]. Notably, several reports have pointed out that muscle stem cells should be considered as a therapeutic target for restoring muscle function in individuals suffering from muscular dystrophies [10,11]. Hence, research to restore SCs function has attracted considerable interest in recent years with the aim of developing new strategies to treat neuromuscular degenerative disorders [11]. 

MicroRNAs (miRNAs) are small, non-coding RNAs that operate post-transcriptionally by interacting directly with 3′untranslated region (3′UTR) of target mRNAs to induce mRNA degradation and translational repression [12]. However, interaction of miRNAs with other regions, including the 5′UTR, coding sequence, and gene promoters, have also been reported [13]. In addition, under certain conditions, miRNAs can also activate translation or up-regulate transcription [14,15,16]. A broad range of miRNAs expressed in the muscle have been shown to play important modulatory roles in a variety of skeletal muscle processes [17]. However, only a limited number of miRNAs have been linked to the regulation of skeletal muscle re-generation and/or satellite cell behavior. Recent evidences have pointed out that miRNAs will contribute to broadening our understanding of controlling factors for skeletal muscle function, as well as to im-proving the understanding and application of current therapeutic strategies in skeletal muscle diseases. In this review, we summarize the latest research achievements of the potential of miRNAs as powerful molecular tools for regulating satellite function in the context of muscle regeneration and muscular disorders such as DMD.

## 2. Muscle Regeneration

**Figure 1:**
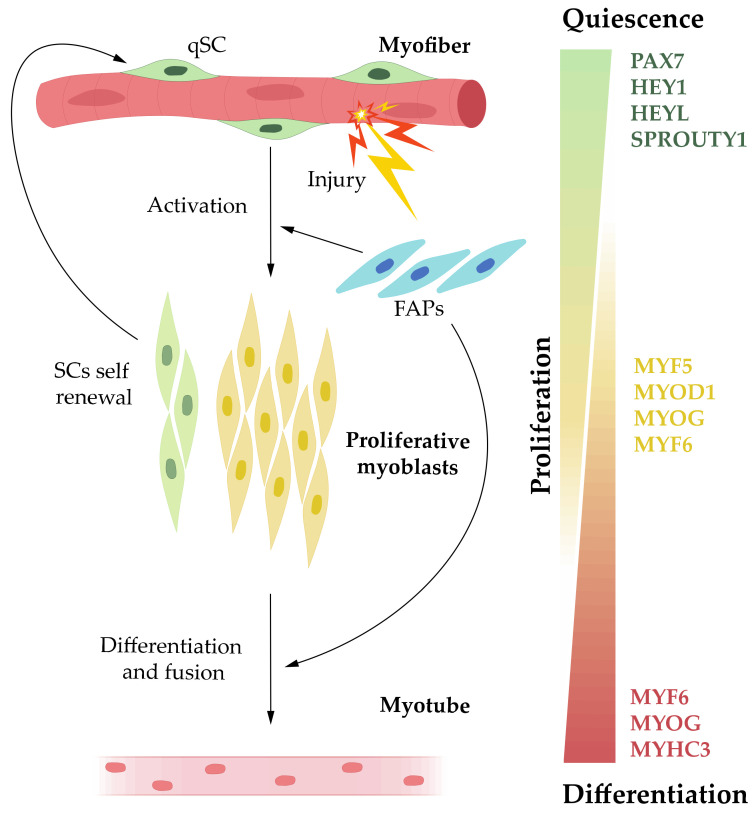
Main phases of myogenesis in regenerative skeletal muscle after injury, including mainly transcription factors and the myogenic regulatory factors (MRFs) involved. SCs, satellite cells; qSC, quiescent satellite cell; FAPs, fibro-adipogenic progenitors.

Muscle regeneration is an important homeostatic process of the adult skeletal muscle that recapitulates many aspects of embryonic myogenesis. Prenatal development is re-activated for muscle reconstruction after injury since both share common key elements, such Pax genes and myogenic regulatory factors (MRFs). SCs in adult muscle represent a lineage continuum of the embryonic myogenic PAX3pos/PAX7pos progenitor cells, which remain in the adult muscle in a quiescent state. SCs are located between the myofiber basal lamina and the sarcolemma of the muscle fiber [18], and they are characterized by PAX7 expression (Figure 1), as well as PAX3 expression in certain muscles. The maintenance of the quiescent state of SCs depends, at least in part, on canonical Notch signals and their target proteins HEY1 and HEYL, as well as on SPROUTY1, a negative regulator of tyrosine kinase signaling [19,20,21,22] (Figure 1). However, some SCs may enter on a G (alert) state, which primes them for rapid entry into the cell cycle, e.g., in response to injury [23].

After damage, SCs activate, proliferate and enter into the myogenic program via PAX7 downregulation and the sequential activation of the myogenic regulatory factors MYF5, MYOD1, MYOG, and MYF6 to generate either myogenic progenitors (myoblasts) that fuse to existing myofibers or de novo myofibers (Figure 1). Activated SCs undergo symmetric and asymmetric divisions. Asymmetric divisions generate both myogenic committed progenies (PAX7pos/MYF5pos cells) and an offspring that maintains their stem cell identity (PAX7pos/MYF5neg cells), whereas symmetric expansion only helps to re-plenish the stem cell pool (PAX7pos/MYF5neg) [6]. Thus, the first myogenic regulatory factor (MRF) to be expressed in the activated SCs is MYF5; the sequential upregulation of Myf5, followed by Myod1, is required for myogenic determination [6]. Activated and committed SCs migrate to the site of injury, and either fuse with damaged myofibers or become myogenic progenitor cells. The migration of SCs is controlled by signals from the myo-fibers, including signaling through EPHRIN and WNT7A [24,25].

Terminal differentiation is initiated by the expression of MYF6 and MYOG [6] (Figure 1). MYOG is located downstream of MYOD1, and represents a key factor in myoblast fusion, as it triggers the expression of contractile proteins, such as myosin light chain, myosin heavy chain and troponin, inter alia [26]. At this point, MYOD1 expression levels are reduced, while contractile proteins start their expression and myocytes fuse to form new multinucleated myotubes or join to damaged myofibers [5]. The new formed myofibers are characterized by centrally located nuclei and the expression of MYHC3 protein, which is a myosin heavy chain that is, otherwise, only expressed during embryonic development [27] (Figure 1). This process is followed by maturation into myofibers. In this scenario, an early fusion has been observed, which involves myocyte-myocyte contact, as well as a late fusion, where the myocytes fuse to multinucleated myotubes [5]. Concomitantly, MRF6 expression produces myotube maturation, allowing myofilament reorganization and central nuclei migration. In this way, a correct balance between several MRFs that control cell fate is critical during the regenerative process.

In addition, muscle regeneration comprises the interaction between a set of heterogeneous cells, such as vascular, inflammatory and mesenchymal cells, which cooperate with satellite stem cells to repair damage in a functional cross-talk with the muscle niche. This process starts with an inflammatory response, followed by a necrotic cycle and closure with a regenerative course that leads to a restored tissue [28]. In this context, muscle regeneration is also influenced by other non-myogenic interstitial mesenchymal cells that reside in the extra-cellular matrix (ECM): fibro-adipogenic progenitors (FAPs) (Figure 1). These progenitor cells, whose phenotypical plasticity seems critical during successful muscle repair, are able to differentiate into fibroblasts and adipocytes, but not into myoblasts [29,30]. FAPs are quiescent in healthy muscle and proliferate upon injury. Undifferentiated FAPs can have positive effects on myoblasts, since in vitro and in vivo studies have shown that FAPs can induce the proliferation and differentiation of activated myoblasts [29,31,32]. At the same time, after muscle damage, the differentiation of FAPs into adipocytes is inhibited via the secretion of the proinflammatory signal IL-4 from innate immune cells such as eosinophils, which are chemo-attached to skeletal muscle after damage [33]. Additionally, FAPs are also regulated by growth factors (e.g., hepatocyte growth factor (HGF) and insulin growth factor 1 (IGF-1)) secreted by endothelial cells and M1/M2 macrophages [34]. 

Although FAPs are key regulators of homeostasis and muscle regeneration, these cells are also responsible for fibrosis and chronic inflammation when deregulated under pathological conditions [29,30,35]. Interestingly, under disease or chronic injury, besides differentiating into adipocytes, FAPs contribute to disturbed regeneration by differentiating into fibroblasts, leading to increased fibrosis through the secretion of type I collagen [29].

## 3. MicroRNAs in Muscle Biology

**Figure 2:**
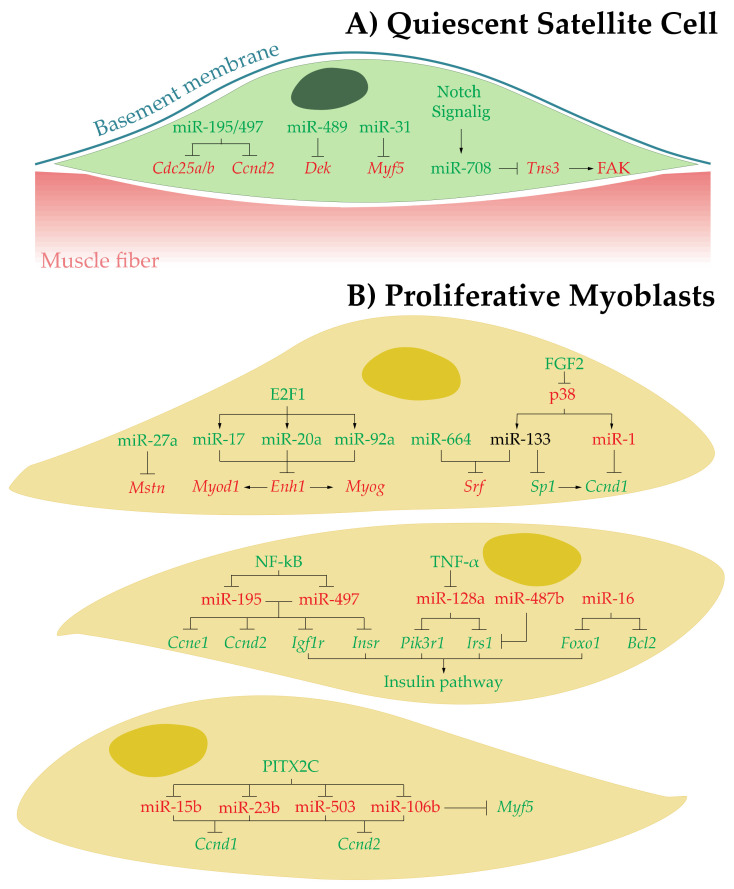
miRNAs network controlling activation and proliferation of muscular precursor cells. (Green and red labels correspond with induced or repressed molecules, respectively).

**Figure 3:**
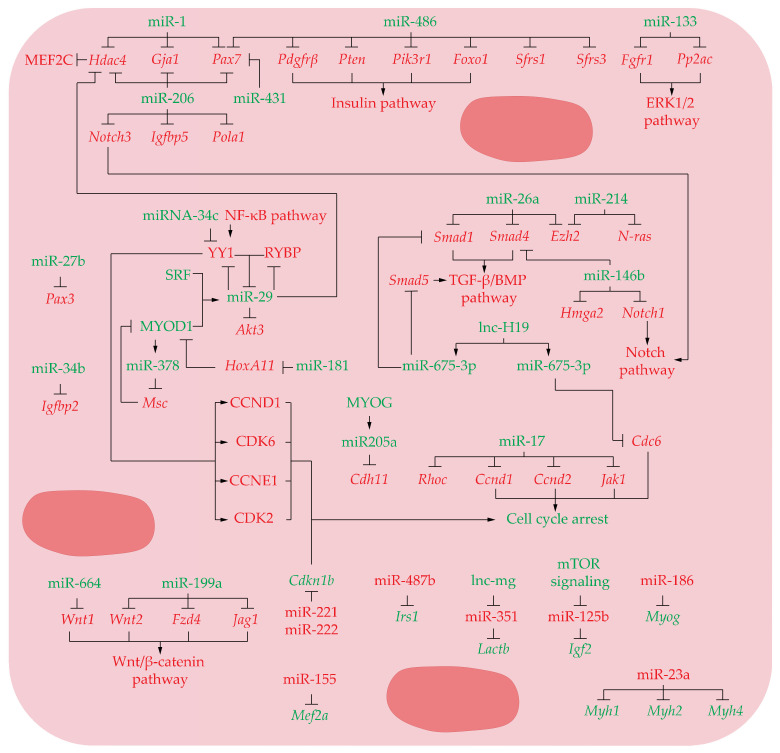
miRNAs network controlling differentiation of muscular precursor cells and myofibers (Green and red labels correspond with induced or repressed molecules, respectively).

MiRNAs play an important role in skeletal muscle development, regeneration, and disease. RNAseq analyses have provided evidences of miRNAs expression during the quiescence, proliferation, and differentiation of muscle precursor cells during myogenesis [36,37,38,39]. Among them, a subset of miRNAs, called myomiRs, have been described either as striated muscle-specific (miR-1, miR-133a, miR-206, miR-208a/b and miR-499) or muscle-enriched (miR-486), being important factors in skeletal muscle myogenesis [40,41,42,43,44]. In addition, others non-muscle-specific miRNAs are also involved in myogenesis and muscle homeostasis by controlling SCs quiescence as well as myoblast proliferation and differentiation processes (Figure 2 and Figure 3; Table 1 and Table 2). Because SCs are the main muscle stem cells responsible for its regenerative capacity, in this section we will analyze the roles exerted by all these miRNAs within the context of SC behavior and function.

### 3.1. MicroRNAs in Quiescent SCs

In the resting muscle fibers, SCs maintain a quiescent state due, at least partially, to miR-195, miR-497, miR-489, miR-31, and miR-708 functions [37,45,46,47,48]. In SCs isolated from different mouse models miR-195 and miR-497 induce cell cycle arrest by targeting cell cycle genes Cdc25a/b and Ccnd2, key elements controlling the cycle progression [45]. MiR-489 is highly expressed in quiescent SCs and suppresses DEK proto-oncogene (Dek), whose protein promotes the transient proliferative expansion of myogenic progenitors [46,49,50]. On the other hand, miR-31 prevents MYF5 protein accumulation [47] and miR-708 represses Tensin3 (Tns3), this repression inhibits FAK activation, which stabilizes SCs within their niche [48] (Figure 2A and Table 1). Altogether, these miRs maintain SCs in an unproliferative state, preventing their premature activation and avoiding their movement.

### 3.2. MicroRNAs in Proliferative Myoblasts

When the muscle regeneration process starts, SCs become activated. Shortly afterwards, a group of molecular signals collaborate in order to enhance and/or promote myoblast proliferation, either by inducing the expression of miRs that target proliferation inhibitors or avoiding repression exerted by several miRs over pro-proliferative key molecules.

#### 3.2.1. Upregulated miRNAs

Within the first group, in vitro analysis miR-27a promotes myoblast proliferation by targeting Myostatin (Mstn), a critical inhibitor of myoblasts proliferation and differentiation [51,52] (Figure 2B and Table 1). Besides, additional in vitro and in vivo experiments in mice showed that the transcription of miR-17-92 cluster is activated in proliferating myoblasts by E2F1 factor thus allowing miR-17, miR-20a, and miR-92a to repress the actin-associated protein enigma homolog 1 (Enh1), a protein that promotes the expression of Myod1 and Myog mRNAs. Hence, ENH1 inhibition represses myoblasts differentiation maintaining the proliferative capability [53]. miR-664 and miR-133 also induce a proliferative state in myoblasts by targeting serum response factor (Srf), a transcription factor that activate cell cycle genes [54,55,56] (Figure 2B and Table 1). At this point, it is interesting to highlight that the role exerted by miR-133 in the proliferative phase still remains controversial, since it has been shown that miR-1/133 targets Sp1/Cyclin D1 in myoblasts and this miRs-mediated repression must be inhibited by FGF2/p38 signaling to enter in a proliferative state [57] (Figure 2B and Table 1). Since, miR-133 opposite effects on myoblasts proliferation have been observed in vitro and using established cell lines, additional experiments by using in vivo mammalian animal models could help to solve such controversy.

#### 3.2.2. Downregulated miRNAs

In vitro analysis by using C2C12 myoblast cell line demonstrated that myoblasts proliferation is induced partially by miR-195 and miR-497 repression mediated by proliferative inductors such as NF-κB [58,59,60], leading to an increase of miR-195-miR-497 targets: the cell cycle genes Cyclin E1 (Ccne1), and Cyclin D2 (Ccnd2), and the mitogens Insulin-like growth factor I receptor (Igf1r) and Insulin receptor (Insr) [61]. Motohashi et al. showed that, the inhibitory effect exerted by the pro-inflammatory cytokine TNF-α [62,63,64,65,66] on miR-128a expression, which targets key elements within Insulin signaling pathway such as Insulin receptor substrate 1 (Irs1) and Phosphoinosi-tide-3-kinase regulatory subunit 1 (Pik3r1), also facilitates myoblasts divisions and miR-128 inhibition in mice lead to increase skeletal muscle mass and fiber size [67] (Figure 2B and Table 1). In this regard, the fundamental role of the miRNAs-Insulin signaling pathway in maintaining myoblast proliferation has also been corroborated through additional in vitro experiments per-formed by Wang et al., who later showed how miR-487b must be downregulated during SCs proliferation in order to maintain high levels of its direct target Irs1 [68] (Figure 2B and Table 1). Jia et al. also showed that another important element in the Insulin signaling pathway, Foxo1, is a target for miR-16, and miR-16 downregulation is required to maintain FOXO1 protein expression in proliferating myoblasts [69,70]. 

On other hand, miR-16 also targets Bcl2 (Bcl2), an important component of the apoptotic and focal adhesion pathways that play an essential role in primary chicken myotube formation [69,70]. Hence, miR-16 downregulation promotes myoblast proliferation and inhibits myoblasts apoptosis coordinately [71] (Figure 2B and Table 1). In the framework of myoblast proliferation and differentiation, our group have also reported that the c-isoform of the transcription factor Pitx2 increases cell proliferation in myoblasts by downregulating miR-15b, miR-23b, miR-106b and miR-503, thus allowing their targets Ccnd1 and Ccnd2 to contribute to myoblasts proliferation in mice [72]. In addition, this Pitx2c-miRs pathway also regulates cell proliferation in early-activated satellite cells through miR-106b inhibition, thus allowing MYF5 protein to promote SCs commitment to a myogenic cell fate [72] (Figure 2B and Table 1).

**Table 1:** General overview of miRNAs involved in quiescent satellite cells and proliferative myoblast states. Arrows indicate miRs that are expressed (↑) or downregulated (↓) within each cell stage.

microRNAs	Targets	Function	References
**Quiescent Satellite Cell State**			
miR-195/497↑	Cdc25a/b and Ccnd2	Promotes SCs quiescence by inducing cell cycle arrest	[45]
miR-489↑	Dek	Regulates SCs quiescence	[46]
miR-31↑	Myf5	Prevents MYF5 protein accumulation and premature activation of SCs	[47]
miR-708↑	Tns3	Regulates quiescence and self-renewal by active repression of SCs migration	[48]
**Proliferative Myoblast State**			
miR-27 ↑	Mstn	Enhances and/or promotes myoblast proliferation	[51]
miR-17, miR-20a, and miR-92a↑	Enh1	Enhance and/or promotes myoblast proliferation	[53]
miR-664↑ and miR-133↑	Srf	Enhances and/or promotes myoblast proliferation by inducing cell cycle genes expression	[54,55]
miR-133↓	Sp1	Inhibit myoblast proliferation	[57]
miR-1↓	Ccnd1	Inhibit myoblast proliferation	[57]
miR-195 and miR-497↓	Igf1r, Insr, Ccne1 and Ccnd2	Inhibit myoblast proliferation	[61]
miR-128a↓	Irs1 and Pik3r1	Inhibits myoblast proliferation	[67]
miR-487b↓	Irs1	Inhibits myoblast proliferation	[68]
miR-16↓	Foxo1 and Bcl2	Inhibits myoblast proliferation and promotes myoblast apoptosis	[71]
miR-15b, miR-23b, miR-106b, and miR-503↓	Ccnd1 and Ccnd2	Inhibits myoblast proliferation	[72]
miR-106b↓	Myf5	Prevents commitment to myogenic cell fate	[72]

### 3.3. MicroRNAs in Differentiating Myoblast

#### 3.3.1. Upregulated miRNAs

The switch from proliferation to differentiation is critical for skeletal myogenesis. During the differentiation stage, the myoblasts need to turn off proliferative signals and upregulate structural genes in order to convert simple individual cells into a complex syncytium with the ability to coordinately contracts. During the last decade many miRs have been identified as related with this process. Thus, under physiological conditions, miR-1 promotes myoblast differentiation by targeting histone deacetylase 4 (Hdac4) mRNA, a transcriptional repressor of myocyte enhancer factor 2C (MEF2C), an essential muscle-related transcription factor [54,73] (Figure 3 and Table 2). In addition, in vivo analysis during muscle development in mice together in vitro experiments revealed that miR-1 and miR-206 restricts myogenic progenitor cell proliferation and promotes differentiation by directly downregulating gap junction protein alpha 1 (Gja1, also known as connexin 43) and Pax7 mRNA [17,74,75,76] (Figure 3 and Table 2). In addition, miR-206 also enhances myoblast differentiation by targeting important inhibitors of myogenesis, such as Hdac4, Notch receptor 3 (Notch3) and insulin-like growth factor binding protein 5 (Igfbp5), and inducing cell cycle arrest through the repression of DNA polymerase alpha 1 catalytic subunit (Pola1), a specific subunit of DNA polymerase α [76,77,78] (Figure 3 and Table 2). Together with miR-206 and miR-1, in vitro and in vivo loss and gain of function experiments have shown that miR-431 and miR-486 also promote myoblast differentiation by targeting Pax7 [75,79]. MiR-486 also exerts this effect by targeting platelet-derived growth factor receptor β (Pdgfrβ) and several components of the PTEN/AKT pathway such as phosphatase and tensin homolog (Pten), Pik3r1 and Foxo1, along with splicing factors, namely, splicing factor arginine/serine-rich 1 (Sfrs1) and splicing factor argi-nine/serine-rich 3 (Sfrs3) [80] (Figure 3 and Table 2). This reinforces the importance about the role of Insulin-related pathways not only in proliferating myoblasts but also in differentiating myotubes. 

Another miR of relevance in this scenario is miR-133. This microRNA promotes myoblasts differentiation though targeting fibroblast growth factor receptor 1 (Fgfr1) and protein phosphatase 2A catalytic subunit, alpha isozyme (Pp2ac) thus indirectly re-pressing the ERK1/2 signaling, a key pathway that enhance myoblast proliferation and block differentiation [81,82] (Figure 3 and Table 2). As revealed by in vivo and in vitro analysis in mice, miR-27b is also expressed in differentiating myoblasts, reducing my-oblast proliferation and ensuring rapid and robust entry into the myogenic differentiation program by targeting Pax3, a fundamental transcription factor that maintain myogenic precursors in an undifferentiated stage [83,84] (Figure 3 and Table 2).

In pre-differentiating myoblasts, miR-29 expression is repressed NF-κB-YY1 transcription factor (Yy1) pathway along RING1- and YY1-binding protein (Rybp) [85,86]. However, as differentiation ensues, the recruitment of the MYOD1/SRF complex displaces the RYBP/YY1 repressive complex leading to the activation of miR-29 promoter. After that, miR-29 targets both Rybp and Yy1 mRNAs through a negative feedback, thus ensuring the progress of myogenic differentiation [85,86]. Concomitantly, miR-29 also balance proliferation vs differentiation of myoblasts by targeting the proliferative factor AKT serine/threonine kinase 3 (Akt3) and promoting differentiation by targeting Hdac4 together with miR-1 and miR-206 [77,87] (Figure 3 and Table 2). 

It has been shown that, during muscle regeneration in mice, miR-26a promotes myoblast differentiation by targeting the transcription factors SMAD family member 1 (Smad1) and SMAD family member 4 (Smad4) [88]; two critical members of the TGF-β/BMP pathway implicated in maintain muscle stem cells pool by preventing myogenic differentiation [88,89]. Moreover, miR-26a also targets the histone methyl-transferase enhancer of zeste homolog 2 (Ezh2), a negative regulator of myogenesis [90,91]. This repressive effect is emulated by miR-214 which also targets Ezh2 mRNA transcript, thus inducing the same impact in muscle cell differentiation [92]. miR-214 likewise contributes to promote myogenic differentiation by facilitating the exit from mitosis through target the proto-oncogene N-ras [93] (Figure 3 and Table 2). At the same time, MiR-146b has also been shown to promote myoblast differentiation pathway by targeting the Smad4 transcripts [94]. Concomitantly, miR-146b also target notch receptor 1 (Notch1) and high mobility group AT-hook 2 (Hmga2) transcripts [94]. Because Notch signaling pathway prevents myoblast differentiation by maintaining muscle stem cell quiescence [22,94,95,96] and HMGA2 interrupts differentiation keeping myoblasts in a proliferative state [97], miR-146b activity strongly facilitates myogenic differentiation [94] (Figure 3 and Table 2).

Several Long Non-Coding RNAs (lncRNAs) have been identified as regulators of development and homeostasis of the skeletal muscle [98,99]. Interestingly, two conserved micro-RNAs, miR-675-3p and miR-675-5p encoded on exon1 of lnc-H19 enhance myoblast, differentiation, miR-675-3p by targeting Smad1 and SMAD family member 5 (Smad5) members of the BMP pathway, whereas miR-675-5p represses the DNA replication by targeting initiation factor cell division cycle 6 (Cdc6) [99] (Figure 3 and Table 2).

MiR-17 collaborates to promote the switch from proliferation to differentiation as well. This miR repress cycle progression by targeting Ccnd1, Ccnd2, and the Janus kinase 1 (Jak1), a key kinase in the JAK–STAT signaling pathway that exerts a marked effect on myoblast proliferation [100,101,102]. Finally, miR-17 also facilitates myotube formation in-creasing extracellular matrix expression, required for cell fusion, by targeting the ras homolog Rhoc, a small signaling G protein of the Rac subfamily that suppresses cell motility and regulates cell fusion [100,103,104] (Figure 3 and Table 2). In addition, two miR-34 family members, miR-34b and miR-34c, are also involved in cell cycle arrest and muscle differentiation progression. In myoblasts, miR-34b suppress the cell cycle progression and accelerate the development of myotubes by targeting the insulin-like growth factor binding protein 2 (Igfbp2), which stimulates myoblasts cell proliferation but suppress myotube formation [105,106]. Similarly, miRNA-34c inhibits myoblast proliferation by targeting CCND1, CDK6, CCNE1, CDK2 and increases differentiation by inhibiting the repressor of myogenesis Yy1 transcription factor [105,106,107] (Figure 3 and Table 2).

Other miRNAs are potent inductors of myogenesis, miR-181 is strongly upregulated during differentiation and participates in establishing the muscle phenotype by targeting the homeobox protein Hox-A11 (HoxA11), a negative regulator of Myod1 expression [108]. Regarding this, it has been described that MYOD1 protein is able to bind in close proximity to miR-378 gene locus, thus causing its transactivation [109]. Concomitantly, this miR targets Musculin (Msc) mRNA, also known as MyoR, thus abolishing the antagonist effect of MSC over MYOD1 and, hence, promoting myogenic differentiation by a feed-forward loop where MYOD1 indirectly downregulates Msc via miR-378 [109] (Figure 3 and Table 2). In the course of myogenic differentiation, miR-205a expression is up-regulated by MYOG transcription factor [110] and miR-205a can inhibit myoblast proliferation and promotes myoblast differentiation by targeting cadherin 11 (Cdh11) mRNA, a strong inhibitor of myoblast differentiation [110] (Figure 3 and Table 2). Finally, several studies have indicated that the Wnt/β-catenin signaling pathway is responsible to inhibit myogenic differentiation [55,111]. It has been shown that this pathway can be precisely down regulated by miR-664, which target Wnt family member 1 (Wnt1) and miR-199a that modulates Wnt family member 2 (Wnt2), frizzled class receptor 4 (Fzd4) and jagged canonical Notch ligand 1 (Jag1), thus allowing to myoblast differentiation [55,111] (Figure 3 and Table 2).

#### 3.3.2. Downregulated miRNAs

All miRs described above must be upregulated during muscle differentiation. Nonetheless, other miRNAs need to be downregulated in order to prevent their sup-pressive effect over their targets, thus promoting the cell cycle exit and /or myoblast differentiation. In this sense, when myoblast differentiation starts miR-221 and MiR-222, which target the cell cycle inhibitor cyclin-dependent kinase inhibitor 1B (Cdkn1b), also known as P27, they are downregulated, thus allowing P27 upregulation and cell cycle arrest [112] (Figure 3 and Table 2). In myogenic cells, miR-351 targets lactamase-β (Lactb), an inhibitor of myoblast proliferation [113]. MiR-487b suppresses the proliferation and differentiation of myoblasts in vitro by targeting Irs1 in skeletal muscle myogenesis. Hence, its downregulation is also mandatory to reach myoblast differentiation [68] (Figure 3 and Table 2). In order to prevent miR-351-5p effect, the long noncoding RNA lnc-mg acts as a competing endogenous RNA (ceRNA) for this miRNA reducing its effect Lactb and promoting the progression toward myoblast differentiation [113,114]. This highlights the versatility of roles played by lncRNAs in the control of the myogenesis process, being able not only to be miR precursors but also acting as miR inhibitors.

Several miRNAs negatively modulate myoblast differentiation, miR-155 represses Mef2a mRNA, and its downregulation is necessary to prevent Mef2a down-regulation and to induce proper myoblast differentiation in C2C12 cells [115] (Figure 3 and Table 2). MiR-125b targets insulin-like growth factor 2 (Igf2), an embryonic regulator of myo-genesis and an autocrine factor that initiates myoblast differentiation in vitro [116,117]. During myoblasts differentiation, Kinase-independent mTOR signaling is able to induce miR-125b repression, thus allowing IGF2 protein to promote myoblast differentiation [118] (Figure 3 and Table 2).

As we mentioned in previous sections, once activated, the expression of Myog allows to the differentiating myoblasts to undergo terminal myogenesis and fuse to form myo-fibers. In this regard, it has been shown that miR-186 inhibits myoblast differentiation by targeting Myog. Hence, miR-186 downregulation is also needed to promote in vitro myoblast differentiation [119] (Figure 3 and Table 2).

Finally, miRs are also capable to regulate structural proteins needed at last stages of myoblast differentiation. Regarding this, it has been shown that miR-23a prevents myogenic differentiation through downregulation of fast myosin heavy chain isoforms in cultured myoblats. Therefore, downregulation of miR-23a during the final steps of muscle differentiation allows myotubes to express the myosin heavy chain genes Myh1, Myh2, and Myh4 [120] (Figure 3 and Table 2).

Overall, all these works have pointed out the relevance to miRNAs in satellite cell biology highlighting how the dynamic and coordinated expression of different miRNAs can orchestrate the response to SC during muscle regeneration modulating the three-key step such as the switch from quiescence to activation, proliferation, and differentiation. Then, miRNAs may be used as very valuable tools to modify different and specific aspects of the satellite cell function.

**Table 2:** General overview of miRNAs involved in differentiating myoblasts state. Arrows indicate miRs that are expressed (↑) or downregulated (↓) within each cell stage.

Differentiating Myoblasts State			
miR-1↑, miR-206↑, and miR-29↑	Hdac4	Promotes myoblast differentiation	[54,77]
miR-1↑ and miR-206↑	Gja1	Promote myoblast fusion	[74]
miR-1↑ and miR-206↑	Pax7	Promote satellite cell differentiation and restrict their proliferative potential	[17]
miR-206↑	Pax7	Activates myoblast differentiation	[75]
miR-206↑	Pax7, Notch3, and Igfbp5	Stimulates SC differentiation and skeletal muscle regeneration	[76]
miR-206↑	Pola1	Promotes myoblast differentiation by inducing a cell cycle arrest	[78]
miR-486↑	Pax7	Promote initial muscle differentiation	[75]
miR-486↑	Pdgfrβ, Pten, Pik3r1, Foxo1, Sfrs1, and Sfrs3	Promotes myoblast differentiation by inhibiting PTEN/AKT pathway and splicing factors	[80]
miR-431↑	Pax7	Mediates satellite cell heterogeneity and promotes muscle differentiation	[79]
miR-133↑	Fgfr1 and Pp2ac	Promotes muscle precursor cells differentiation	[82]
miR-27b↑	Pax3	Ensures rapid and robust entry into the myogenic differentiation program	[83]
miR-29↑	Rybp and Yy1	Ensures proper myoblast differentiation into myotubes	[85,86]
miR-29↑	Akt3	Reduces proliferation and facilitate differentiation of precursor muscle cells	[87]
miR-26a↑	Smad1 and Smad4	Promotes myoblast differentiation	[88]
miR-26a↑ and miR-214↑	Ezh2	Induces muscle cell differentiation	[91,92]
miR-214↑	N-ras	Promotes myogenic differentiation by facilitating exit from mitosis	[93]
miR-146b↑	Smad4, Notch1, and Hmga2	Promotes myogenic differentiation	[94]
miR-675-3p↑	Smad1 and Smad5	Promotes myogenic differentiation downregulates the BMP pathway	[99]
miR-675-5p↑	Cdc6	Promotes myogenic differentiation by repression of DNA replication	[99]
miR-181↑	HoxA11	Promotes myogenic differentiation	[108]
miR-378↑	Msc	Promotes myogenic differentiation	[109]
miR-205a↑	Cdh11	Inhibits myoblast proliferation and promote myoblast differentiation	[110]
miR-17↑	Ccnd1, Ccnd2, Jak1, and Rhoc	Promotes differentiation of precursor muscle cells by inducing cell cycle arrest and extracellular matrix expression	[100]
miR-34b↑	Igfbp2	Represses proliferation and promotes differentiation of myoblasts	[106]
miR-34c↑	Yy1	Represses proliferation and promotes differentiation of myoblasts by leading to G0/G1 arrest	[107]
miR-664↑	Wnt1	Downregulates Wnt/β-catenin signaling pathway to allow normal myogenic differentiation	[55]
miR-199a↑	Wnt2, Fzd4, and Jag1	Downregulates Wnt/β-catenin signaling pathway to allow normal myogenic differentiation	[111]
miR-155↓	Mef2a	Represses myoblast differentiation	[115]
miR-487b↓	Irs1	Suppresses the proliferation and differentiation of myoblasts by repressing PI3K/Akt and MAPK/Erk pathways	[68]
miR-221 and 222↓	Cdkn1b	Inhibits myoblast differentiation by allowing myoblast to proliferate and avoiding to acquire myotube morphology	[112]
miR-351↓	Lactb	Represses myoblast differentiation	[113]
miR-125b↓	Igf2	Represses myoblast differentiation	[118]
miR-186↓	Myog	Represses myoblast differentiation	[119]
miR-23a↓	Myh1, Myh2, and Myh4	Prevents myogenic differentiation by inhibiting myosin genes expression	[120]

## 4. Regulatory Role of miRNAs in Muscle Regeneration as a Therapeutic Target in DMD

Muscular dystrophies are the most important group among primary muscular dis-orders in terms of number of people affected as well as economic impact generated. These pathologies are inherited myogenic disorders characterized by progressive muscle wasting and weakness of variable distribution and severity [121]. Duchenne Muscular Dystrophy (DMD) is the most common inherited muscle diseases in childhood, affecting 1 in 3500 live male births [122,123,124]. DMD is a muscular disorder caused by mutations in the dystrophin gene, located in the short arm of the X chromosome. The absence or defects in DYSTROPHIN protein results in chronic inflammation, progressive muscle degeneration, and replacement of muscle with fibroadipose tissue [122,123,124]. DMD patients often lose independent ambulation by the time they reach 13 years of age, and generally die of respiratory failure in their late teens or early twenties [124]. In this context, it is important to highlight that although preliminary results for gene replacement in muscular dystrophies are very promising, this therapy involves several disadvantages, as it does not incorporate the full protein into the muscle, together with the potential loss of gene therapy effectiveness over time, making it necessary to develop other synergistic therapeutic approaches that help muscle endurance.

Regarding this, the loss of regenerative potential is a common feature present in DMD [125]. In this muscle dystrophy, the degenerative processes are associated to the loss of proper muscle regeneration, mainly due to muscle stem cells intrinsic defects related to their activation, proliferation, self-renewal, and differentiation contributing to worsen the dystrophic phenotype [6,126]. Nonetheless, how the genetic defect that generates this pathology alters muscle stem cell behavior and muscle regeneration is partially under-stood. Parallel to SCs defects, fibrosis has special relevance in DMD, where the re-placement of muscle with fibroadipose tissue is a major pathological hallmark, thereby contributing to worsen the dystrophic phenotype [122]. In this framework, it is interesting to highlight that the analysis of muscle biopsies of DMD patients as well as the use of DMD cell lines and animal models have provided a significant list of dysregulated miRNAs in DMD [127,128], aiming miRNAs levels restoration as a target to improve disease phenotype. In this section we focus on dysregulated microRNAs impacting SCs behavior and fibrosis in the context of DMD, highlighting different technical approaches implemented in order to avoid such misregulation (Table 3). 

Interestingly, miR-29 is down-regulated in DMD and its overexpression in DMD myoblasts significantly decreases the miR-29 targets described above finally promoting muscle regeneration [129,130]. Moreover, intramuscular and/or intravenous injection of miR-29 in mdx mice, the most widely used animal model for DMD research, promotes muscle function and inhibits fibrogenesis by targeting extracellular matrix components such as collagens and microfibrillar-associated protein 5 (Mfap5) [129,130] (Table 3). Moreover, micro-dystrophin gene delivery together with miR-29 overexpression in mdx/utrn^-/-^ mice (a mouse strain that exhibit a more severe dystrophic phenotype) suppresses fibrosis, restores muscle function, and increases absolute and specific force [131] (Table 3). On other hand, in addition to the miR-206 effect on myoblast differentiation mentioned above, Bulaklak et al showed that the AAV-mediated inhibition of miR-206, another miR dysregulated in DMD, in the muscles of dystrophic mdx mice increases the expression of compensatory “booster genes” in DMD, such as VEGFA and utrophin, thus improving motor function and reducing muscle fibrosis [132] (Table 3). 

Other microRNAs have been shown to be also dysregulated in DMD, for instance, miR-21 expression is increased in DMD muscle biopsies and skeletal muscle fibroblasts [130]. MiR-21 targets PTEN, activating the AKT pathway and thus leading to increased fibrosis [130,133]. Interestingly, it has been reported that miR-21 inhibition in mdx mice can reduce fibrosis in the diaphragm [130]. Besides, miR-21 is also increased concomitantly with age-dependent fibrogenesis, and miR-21 biogenesis is also involved in the PAI-1/plasmin system, regulating ECM remodeling and fibrosis progression [130,133]. Hence, miR-21 inhibition in the muscles of senescent mdx mice can reduce fibrosis and improve muscle homeostasis [133] (Table 3). Therefore, treatment by using miR-21 inhibitors would reduce fibrosis in DMD, slowing down disease progression and thus facilitating the period for muscle regeneration treatment therapies. Additionally, miR-146a has been proposed as a target associated to inflammation and fibrosis in the context muscle dystrophy. miR-146a levels are increased in the muscles of dystrophic mice and it is known that miR-146a can reduce inflammation and fibrosis by down-regulating the expression of proinflammatory cytokines (IL-1β, CCL2, TNFα) [134] (Table 3). miR-31 is also highly expressed in dystrophic mdx mice and in muscle biopsies from DMD patients, and it has been shown to target 3’UTR-dystrophin [135]. Interestingly, it has been re-ported that the combinatory effect of miR-31 inhibition and exon skipping in human DMD myoblasts increase dystrophin synthesis [135] (Table 3). Since miR-31 also targets myogenic factor Myf5 and its down-regulation leads to SCs activation and differentiation [47], we can speculate that the inhibition of miR-31 could be a strong candidate to be proposed as a therapeutic tool to improve both muscle differentiation and dystrophin restoration.

Finally, other miRNAs, that have not been previously shown to be dysregulated in muscular disorders, can also modulate muscle regeneration in the dystrophic context. Regarding this, Wu et al. have observed that in vivo miR-431 overexpression in dystrophic mdx mice accelerated muscle regeneration [79] (Table 3). In addition, it has been de-scribed that overexpression of miR-127 in mdx mice promotes myogenic differentiation and ameliorates the dystrophic phenotype [136] (Table 3).

**Table 3:** Experimental strategies assayed to modulate miRNAs expression in the context of DMD in order to improve SCs behavior and fibrosis.

Muscle Regeneration Event	Target microRNA	Molecular Approach	Experimental System	Reference
Duchenne Muscular Dystrophy				
Fibrosis and Inflammation	miR-29	miRNA Mimic	Human DMD myoblast	[130]
		miRNA Mimic	mdx mice	[129]
		miR-29 + micro-dystrophin overexpression by AAV	mdx/utrn^+/−^ mice	[131]
	miR-206	AntagomiR Sponge	mdx mice	[132]
	miR-21	AntagomiR	Human DMD fibroblasts Mdx mice	[130]
		AntagomiR	Senescence mdx mice	[133]
	miR-146a	KO	mdx mice	[134]
Muscle differentiation	miR-29	miRNA Mimic	Human DMD myoblast	[130]
		miRNA Mimic	mdx mice	[129]
	miR-31	AntagomiR or Sponges + exon skipping	Human DMD myoblasts	[135]
	miR-431	Transgenic overexpression	mdx mice	[79]
	miR-127	siRNA mimic Transgenic overexpression	C2C12 myoblasts mdx mice	[136]

### Current and Future Aplicable Technologies for miRNAs Modulation in DMD

In spite of all these promising results and, in order to develop a proper microRNA-based therapy, different molecular tools could be used. Thus, if we would need to enhance miRNA expression, miRNA replacement can be conducted by introducing a miRNA mimic product. The miR-Mimic technology utilizes synthetic, modified oligo-nucleotides that can bind to the unique sequence of target genes (mRNAs) in a gene-specific manner and elicit post-transcriptional repressive effects as an endogenous miRNA does [137]. On the other hand, to down-regulate microRNAs, their inhibition can be induced by using several miRNA-inhibitory products known as antimiRs. As in the case of miRNA mimic products, antimiRs comprise numerous classes of chemically modified oligonucleotides and nucleic acid analogs, such as locked nucleic acids (LNAs), 2’-O-methyl (2’-O-Me) oligos, 2’-O-methoxyethyl (2’-O-MOE) oligos, antagomiRs, peptide nucleic acids (PNAs) and phosphorodiamidate morpholino oligomers (PMOs) [138]. These chemical modifications are implemented to provide resistance to cellular nucleases and to increase affinity towards complementary miRNA sequences [139,140]. In addition, some antimiRs have flanking sequences or are connected to lipids through the use of linkers [141]. A common technical issue that is present when researchers try to properly deliver all these kind of naked nucleic acids molecules into cell cytoplasm is how to cross trough the cell membrane. In this sense, although the delivery of naked nucleic acids molecules into the cells is considered the safest way of transfection, this process is highly infective due to the electrostatic repulsions occurring at physiological pH between the anionic nucleic acids molecules and the negatively charged plasma membrane [142]. In order to overcome this issue, both miR mimics and anti-miRs are delivered in vitro by using commercially available transfection agents, such as DharmaFECT™ and Lipofectamine™, or by electroporation. Another technical problem is related to the dilution of miR-Mimic and antimiR molecules by successive cell divisions or cytoplasmic metabolism [141]. In order to avoid such dilution, specialized plasmid and virus-vectors carrying expression units for these inhibitory RNA molecules have also been developed [143]. In this sense, novel competitive inhibitors known as ‘miRNA sponges’ are transcripts expressed from strong promoters, containing multiple, tandem binding sites to a miRNA of interest [144]. These miRNA sponge vectors inhibit miRNA function efficiently, although for no longer than one month [145]. This problem has been partially solved by the development of “tough decoy RNAs”, which are 60 base-pair-long hairpin-shaped inhibitors with a large internal bulge containing two miRNA recognition sites [141,145]. These molecules are efficiently exportable to the cytoplasm through plasmid- or lentivirus-based vectors, where their highly potent miRNA inhibitory system persists for over one month [141,145].

However, miRNA-therapy exhibit a number of additional difficulties. On the one hand, naked nucleic acids, even combined with transfection agents, present limited efficacy due to poor undesired off-target [146] or on-target effects [147], short half-life in systemic circulation [148] and limited stability in blood due to their rapid degradation or inactivation by nucleases that are abundantly present in the blood stream [149,150]. On the other hand, viral vectors carry important shortcomings due to their risk of insertional mutagenesis, inherent cytotoxicity and/or immunogenicity [151] tumorigenic risk [152,153]. In addition, viral vectors are prone to entering of undesired cell and tissue types when introduced intravenously or intramuscularly [154]. In order to overcome these technical barriers, new strategies for miR-Mimic and antimiR molecules delivery via nonviral systems such nanoparticles, hydrogels, and exosomes have been developed. 

Inorganic nanoparticles constituted by iron oxides, silica, mesoporous silica, and gold [155,156,157,158,159,160], as well as organic nanoparticles produced by using by synthetic polymers, such poly(ethylene imine)s (PEIs), Poly(lactic-co-glycolic acid) (PLGA), poly(ε-caprolactone) (PCL), and polyurethanes (PUs) [161,162,163,164,165,166] or natural polymers, such hyaluronic acid [167] have been utilized for miRNA delivery, most of them in the cancer field. These nanoparticles present the ability to shield the loaded agent from the external environment, thereby reducing inactivation or degradation, and enhancing circulation time and targeted ac-cumulation [168]. The main challenges in miRNA delivery with nanocarriers are related to their low encapsulation efficiency, and the need for cell targeting [169,170,171]. In this regard, many enforcing attempts have been made in order to improve cell targeting by modifying the nanoparticles surface with ligands for specific receptors present in target cells, thus facilitating its uptake by receptor-mediated endocytosis and reducing the required dosage and side effects of treatment [167,172,173]. However, the nanoparticle circulation time also depends on interactions with the biological microenvironment that could lead to their fast clearance. Regarding this, it has been shown that, once they are exposed to body fluids, the nanoparticle surface is covered by plasma proteins, resulting in masked surface ligands, non-specific uptake, and reduced stability [174,175]. Therefore, nanocarrrier-technology still needs improvements in order to properly drive miRNA to specific cell targets. Within the muscle filed, promising steps have been made in this direction, since nanoparticles functionalized with a muscle-homing peptide M12 have promoted their selective uptake by muscle cells/tissue in vitro and in vivo [176].

Hydrogels are biomaterials originated by self-assembling or crosslinking of water-soluble polymers into a network, thus forming three-dimensional matrices [177]. These porous and hydratable structures induce their gelation and swelling in the biological microenvironment, enabling their local administration by injection without invasive surgery, thus acting as carries that have the ability to take the shape of the corresponding tissue cavity [177]. In order to improve the kinetic release, as well as to preserve the activity of therapeutic biomolecules, hydrogels have been widely investigated as gene delivery systems [178]. There is a great variety of hydrogels depending on their chemical nature. In this regard, either natural-based hydrogels, such alginate, cellulose, chitosan, collagen, dextran, fibrin, pullulan gelatin, and hyaluronic acid, or synthetic hydrogels, as polyethylene-glycol (PEG), poly(N-isopropylacrylamide) (PNIPAm), polyurethane or poly(organophosphazene) have been studied as delivery systems of therapeutic nucleic acids molecules, including microRNAs, in various tissue engineering approaches (reviewed in [179]). Although the controlled delivery of naked microRNAs from hydrogels enhances their local and sustained delivery [180], combination of nanoparticles, as vectors for microRNA delivery, and hydrogels, as delivery media, have shown a strong therapeutic efficacy in the context of heart muscle [181].

Exosomes are extracellular vesicles of endocytic origin secreted by almost every cell type in humans and typically range in 30–100 nm size [154]. These vesicles are able to naturally carry macromolecules such lipids, proteins, and nucleic acids, including microRNAs [182]. Exosomes mediate biological information through direct transfer of intra-exosomal content inside recipient cells by fusion with cell membrane [183], but also by presenting biologically active macromolecules that can selectively bind to cell surface receptors presented by recipient cells [154,184]. Thus, exosomes offer unique advantages over other microRNA delivery systems. First, these vesicles are nonimmunogenic and tend to share characteristics with the host cell from which they are derived [185]. Second, they can deliver miRNA content to specific cell types via receptor-mediated binding, thus being rapidly taken up by recipient cells, minimizing off-target effects exhibited through systemic circulation [154]. In this regard, Sadona et al have recently unraveled an exosome-mediated cross-talk between FAPs and SCs [186]. In this work, they shown how histone deacetylase inhibitors (HDACi), a drug that counters DMD progression by promoting compensatory regeneration, while inhibiting fibro-adipogenic degeneration both in pre-clinical studies and clinical trials [35,187,188,189,190,191], increased levels of miR-206 in exosomes released from FAPs of muscles from Duchenne dystrophic patients or mice [186]. Interestingly, exosomes from HDACi-treated dystrophic FAPs could stimulate SCs activation and expansion ex vivo and promoted regeneration, while inhibiting fibrosis and inflammation of dystrophic muscles, upon intramuscular trans-plantation, in vivo. Altogether, these data reveal a potential for pharmacological modulation of FAP-derived exosomes’ content as novel strategy for focal therapeutic interventions in DMD and possibly other muscular diseases [186]. On the other hand, engineered exosomes could be another highly attractive future alternative for delivering muscle-specific microRNAs in order to modulate DMD pathogenesis. Regarding this, it has been previously shown that cell/tissue-targeting peptides can be fused to the selected exosomal membrane proteins to achieve targeted delivery [192]. Nonetheless, one of the main problems that arises when working with exosomes is to identify the molecular signals that, naturally, are used by the secreting and recipient cells as well as to identify these cells within one specific biological context. All these questions are not easily to address, since exosomes biology still needs an in-depth understanding.

## 5. Conclusions

DMD is a disease caused by a mutation in a gene named dystrophin that gets worse over time, with a severe prognosis, and for which there is currently no cure. The treatments developed in recent years have not been able to effectively improve the disease, except in the case of gene therapy. The earliest results from clinical trials, using a micro-dystrophin gene therapy, were very encouraging. However, this therapy does not cure as it does not incorporate the full protein into the muscle, which still causes muscle damage, such as inflammation. This, together with the potential loss of gene therapy effectiveness over time, creates the need for new treatments that, co-administered with gene therapy, allows the possibility of increasing the regeneration of the damaged muscle. Importantly, in the context of DMD, several recent studies have highlighted that the progressive loss of muscle mass may be attributed, at least partly, to associated defects in muscle regeneration. These intrinsic defects are often linked to the reduced capability of muscle stem cells to generate the appropriate number of myogenic progenitors needed for proper muscle regeneration. In the last decades, it has been shown that miRNAs play a critical role in regulating muscle regeneration and stem cell behavior. Interestingly, some of those miRNAs are altered in the context of muscular dystrophies and can regulate specific molecular targets linked to muscle stem behavior and muscle repair. These findings highlight the importance of using new approaches to manipulate the expression levels of microRNAs in conjunction with gene replacement therapies, in order to improve muscle regeneration in DMD. In this context, the development of new technologies that facilitate miRNA-delivery to muscle stem cells to ameliorate some intrinsic defects linked to dystrophic phenotype will provide us a powerful tool to modulate muscle regeneration in DMD. In the last decade, the development of nanocarrier-technology and/or exosome knowledge may constitute a promising new strategy to improve muscle function in DMD. However, several problems for microRNA therapeutic approaches related to off-target side effects, unwanted toxicity, and specific delivery need to be more deeply addressed.
